# Bivalent structure of a TNFR2-selective and agonistic TNF-α mutein Fc-fusion protein enhances the expansion activity of regulatory T cells

**DOI:** 10.1038/s41598-023-40925-9

**Published:** 2023-08-23

**Authors:** Masaki Inoue, Yuta Tsuji, Reira Ueno, Daisuke Miyamoto, Keisuke Tanaka, Yuka Moriyasu, Saya Shibata, Mei Okuda, Daisuke Ando, Yasuhiro Abe, Haruhiko Kamada, Shin-ichi Tsunoda

**Affiliations:** 1 Laboratory of Cellular and Molecular Physiology, The Faculty of Pharmaceutical Sciences, Kobe Gakuin University, 1-1-3 Minatojima, Chuo-ku, Kobe, 650-8586 Japan; 2 Laboratory of Biopharmaceutical Research, National Institutes of Biomedical Innovation, Health and Nutrition, 7-6-8 Saito-Asagi, Ibaraki, Osaka 567-0085 Japan; 3 Center for Drug Design Research, National Institutes of Biomedical Innovation, Health and Nutrition, 7-6-8 Saito-Asagi, Ibaraki, Osaka 567-0085 Japan; 4 National Institute of Health Sciences, 3-25-26 Tonomachi, Kawasaki-ku, Kawasaki, 210-9501 Japan

**Keywords:** Biologics, Inflammation, Immunotherapy

## Abstract

Recently, TNF receptor type 2 (TNFR2) signaling was found to be involved in the proliferation and activation of regulatory T cells (Tregs), a subpopulation of lymphocytes that suppress immune responses. Tregs mediate peripheral immune tolerance, and the disruption of their functions causes autoimmune diseases or allergy. Therefore, cell expanders or regulators of Tregs that control immunosuppressive activity can be used to treat these diseases. We focused on TNFR2, which is preferentially expressed on Tregs, and created tumor necrosis factor-α (TNF-α) muteins that selectively activate TNFR2 signaling in mice and humans, termed R2agoTNF and R2-7, respectively. In this study, we attempted to optimize the structure of muteins to enhance their TNFR2 agonistic activity and stability in vivo by IgG-Fc fusion following single-chain homo-trimerization. The fusion protein, scR2agoTNF-Fc, enhanced the expansion of CD4^+^CD25^+^ Tregs and CD4^+^Foxp3^+^ Tregs and contributed to their immunosuppressive activity ex vivo and in vivo in mice. The prophylactic administration of scR2agoTNF-Fc suppressed inflammation in contact hypersensitivity and arthritis mouse models. Furthermore, scR2-7-Fc preferentially expanded Tregs in human peripheral blood mononuclear cells via TNFR2. These TNFR2 agonist-Fc fusion proteins, which have bivalent structures, are novel Treg expanders.

## Introduction

Regulatory T cells (Tregs) are a subpopulation of T lymphocytes that have immunosuppressive effects on other immune cells. Tregs exist in mammals such as mice or humans and control immune responses and inflammation. Failure of their functions causes various autoimmune diseases including rheumatoid arthritis (RA) or type I diabetes related to abnormal immune responses to autologous tissues, and various allergies including contact hypersensitivity (CHS) or food allergy as hyperimmune reactions^1,2^. Therefore, expanding Tregs might treat or prevent these immune diseases. Furthermore, increasing the number of Tregs or enhancing their suppressive activity in vivo might suppress rejection reactions in organ transplantation or graft-versus-host disease after hematopoietic stem cell transplantation^3^.

Forkhead box P3 (Foxp3), a transcription factor and master gene in Tregs, controls Treg suppressive functions^4,5^. Tregs exert their immunosuppressive activity by signaling through co-inhibitory receptors including CTLA-4, programmed cell death-1, lymphocyte activation gene-3, or immunosuppressive cytokines including interleukin-10 (IL-10) or transforming growth factor-β (TGF-β)^6–10^. Furthermore, the importance of tumor necrosis factor receptor-2 (TNFR2) signaling for Treg function was revealed recently as it elevated the expressions of CD25 and Foxp3 in Tregs and promoted the proliferation and suppressive activity of Tregs^11^. Tregs express markedly higher levels of TNFR2 than CD4^+^CD25^−^ effector T cells. There is a close relationship between TNFR2 expression and the immunosuppressive functions of Treg cells, and TNFR2 is expressed in a highly suppressive subset of Tregs^11–13^. Moreover, the number and immunosuppressive activity of Tregs in TNFR2^−/−^ mice were markedly lower than in wild-type (WT) mice and WT-Tregs, respectively^14^.

TNF-α is a pleiotropic cytokine that mediates its physiological function via two receptors, TNFR1 and TNFR2. TNFR1 signaling evokes inflammation via an intracellular death domain, which induces cellular apoptosis by activating caspase 8, a cysteine protease^15,16^. TNFR2 has no death domain and its activation upregulates NF-κB via TRAF2 to promote cell growth^17,18^. TNFR1 is expressed ubiquitously by various cells, whereas TNFR2 is limited to specific cells including T lymphocytes, Tregs, endothelial cells, and neural cells. These features suggest TNFR2 might be a promising target molecule for the proliferation and activation of Tregs.

We previously generated TNF-α mutated proteins, R2agoTNF and R2-7, with TNFR2 selective agonistic activity via mouse TNFR2 and human TNFR2, respectively by phage display technology^19–21^. Amino acid replacement in the TNFR interaction region of TNF-α afforded TNFR2 selective binding ability and bioactivity via TNFR2. Therefore, R2agoTNF could expand mouse CD4^+^CD25^+^ Tregs ex vivo, although it had a short half-life in blood because it was rapidly excreted within 24 h. Soluble TNF-α has a high affinity for TNFR1. However, membrane-bound TNF-α has a higher affinity for TNFR2 than TNFR1^22,23^. Therefore, molecules that activate TNFR2 signaling similar to membrane-bound TNF-α might preferentially expand Tregs. We reported that IgG-Fc fusion of single-chain TNFR1-selective antagonistic TNF-α, scR1antTNF-Fc, extended its retention in vivo^24^. scR1antTNF-Fc at low doses was effective in arthritis mice. Therefore, an Fc-fusion protein of R2agoTNF, scR2agoTNF-Fc, might have extended stability in blood. Similar to membrane-bound TNF-α, the bivalent structure of the Fc fusion protein might enhance Treg expansion activity via TNFR2 compared with unmodified R2agoTNF.

Here, we generated scR2agoTNF-Fc and scR2-7-Fc, and characterized their molecular functions. We confirmed the ex vivo Treg expansion activities of scR2agoTNF-Fc and scR2-7-Fc, and effects of scR2agoTNF-Fc on mouse disease models including skin CHS and RA.

## Results

### Molecular characterization of a TNFR2 agonist Fc fusion protein

**Figure 1:**
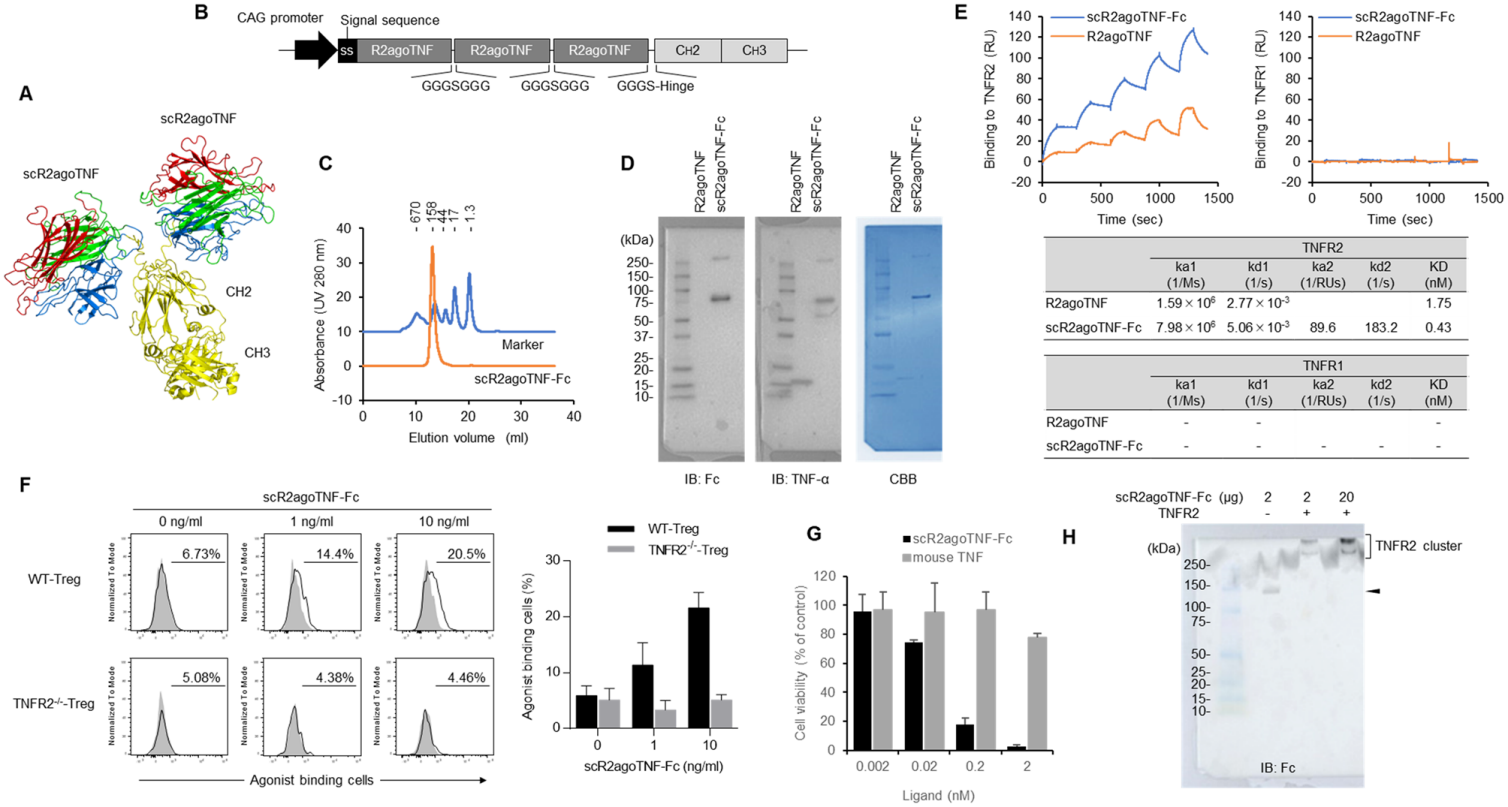
Generation and characterization of scR2agoTNF-Fc protein. (**A**) X-ray structure modeling of the scR2agoTNF-Fc protein. Trimeric structure of scR2agoTNF (each monomer is indicated sequentially by red–green–blue) fused human IgG-Fc (Ch2 and Ch3 are indicated in yellow). (**B**) Schematic pCAG-based mammalian expression of scR2agoTNF-Fc protein. The cDNA was composed of triple R2agoTNF domains fused by peptide linkers (GGGSGGG) that were further fused to a human IgG-Fc domain (Ch2 and Ch3). A signal sequence peptide gene derived from a mouse IgG Vh was linked at the 5′-terminal. (**C**) Isolated recombinant protein (indicated by an orange line) was analyzed by size-exclusion chromatography. Molecular size marker is indicated by a blue line. (**D**) The molecular weight of monomeric scR2agoTNF-Fc was confirmed by western blotting with anti-human IgG-Fc antibody and anti-human TNF-α antibody. After detection, the PVDF membrane was stained by Coomassie brilliant blue. A Precision Plus Protein Kaleidoscope Standards (Bio-Rad) was used for protein size markers. Full-length membrane images with the bandless area cropped are provided. The original blots are presented in Supplementary Fig. S1A. (**E**) In vitro receptor binding activity of scR2agoTNF-Fc or R2agoTNF to TNFR2 and TNFR1 was analyzed by SPR. Sensorgram indicates the association (120 s) and dissociation (120 s) repeats at five serial concentrations (1.2, 3.7, 11.1, 33.3, and 100 nM) using single-cycle kinetics. Kinetic parameters of R2agoTNF and scR2agoTNF-Fc were analyzed with monovalent and bivalent models, respectively, using BIAcore X-100 evaluation software (n = 1). (**F**) Dose-dependent binding levels of scR2agoTNF-Fc to native TNFR2. scR2agoTNF-Fc positive cells in WT-CD4^+^Foxp3^+^ Tregs and TNFR2^−/−^CD4^+^Foxp3^+^ Tregs were examined by FCM. Data are the mean ± SD (n = 3). (**G**) Signal transduction activity via TNFR2 was evaluated by cytotoxic assay. Cytotoxicity of mouse TNF or scR2agoTNF-Fc was measured using mouse TNFR2/Fas overexpressing preadipocytes. (**H**) Clustering with scR2agoTNF-Fc and recombinant mouse TNFR2 was measured by native-PAGE. The protein complex was detected by western blotting with an anti-human IgG antibody. The original blots are presented in Supplementary Fig. S1B.

We developed scR2agoTNF-Fc, consisting of human IgG1-Fc fused to scR2agoTNF protein on the C terminus. The model structure demonstrated that TNFR2 agonist proteins become bivalent by Fc domain via the hinge region (Fig. 1A). The Fc domain was unaffected and scR2agoTNF bound to TNFR2 because the Fc domain was far from scR2agoTNF and did not interfere with the receptor interaction region. Recombinant protein was expressed using mammalian cells. pCAG-based expression vectors were cloned (Fig. 1B) and transfected into Expi293F cells^24^. After cell cultivation for 7 days, scR2agoTNF-Fc protein was purified using immobilized metal ion affinity chromatography and size-exclusion chromatography. The purified protein was detected as a single peak of the expected molecular weight by size-exclusion chromatography (Fig. 1C). SDS-PAGE and western blotting demonstrated this protein had a bivalent structure of 75-kDa monomer composed of scR2agoTNF and human IgG-Fc (Fig. 1D). TNF-α binds to TNFR1 and TNFR2, but R2agoTNF binds only to TNFR2. The binding avidity of scR2agoTNF-Fc for mouse TNFR1 or mouse TNFR2 was assessed by surface plasmon resonance (SPR) (Fig. 1E). The affinity of R2agoTNF and avidity of scR2agoTNF-Fc were evaluated at concentrations of 6.25, 12.5, 25.0, 50.0, and 100 nM by single-cycle kinetics. Both proteins showed dose-dependent responses for TNFR2, but the maximum binding response (Rmax) values of scR2agoTNF-Fc (Rmax: 132.6 RU) were higher than for R2agoTNF (Rmax: 41.3 RU) (Fig. 1E). Of note, neither protein had affinity for TNFR1. The TNFR2 Kd of scR2agoTNF-Fc (0.43 nM) was lower than that of R2agoTNF (1.75 nM) indicating the bivalent structure of scR2agoTNF-Fc enhanced its binding to TNFR2. To confirm the binding capacity to native TNFR2, differences in the binding levels of scR2agoTNF-Fc to CD4^+^Foxp3^+^ WT-Tregs or TNFR2^−/−^Tregs ex vivo were examined by flow cytometry (FCM) (Fig. 1F). We found that scR2agoTNF-Fc bound to WT Treg dose-dependently, but not to TNFR2^−/−^Treg. Therefore, scR2agoTNF-Fc was confirmed to bind to mouse TNFR2. Signal transduction via TNFR2 was assessed by analyzing the viability of mouse TNFR2/Fas-preadipocytes. Cells overexpressing the fusion chimera receptor of mouse TNFR2 and mouse Fas can undergo cell death through TNFR2. The concentration-dependent death of mouse TNFR2/Fas preadipocytes showed scR2agoTNF-Fc-induced signal transduction via TNFR2 (Fig. 1G). scR2agoTNF-Fc co-incubated with TNFR2 recombinant protein was detected as an upper-band compared with TNFR2 protein by undenatured SDS-PAGE (Fig. 1H). Therefore, the bivalent structure of scR2agoTNF-Fc evokes ligand-TNFR2 clusters on the cell surface to enhance TNFR2 signaling.

### Treg proliferation and NF-κB signaling induced by scR2agoTNF-Fc

**Figure 2:**
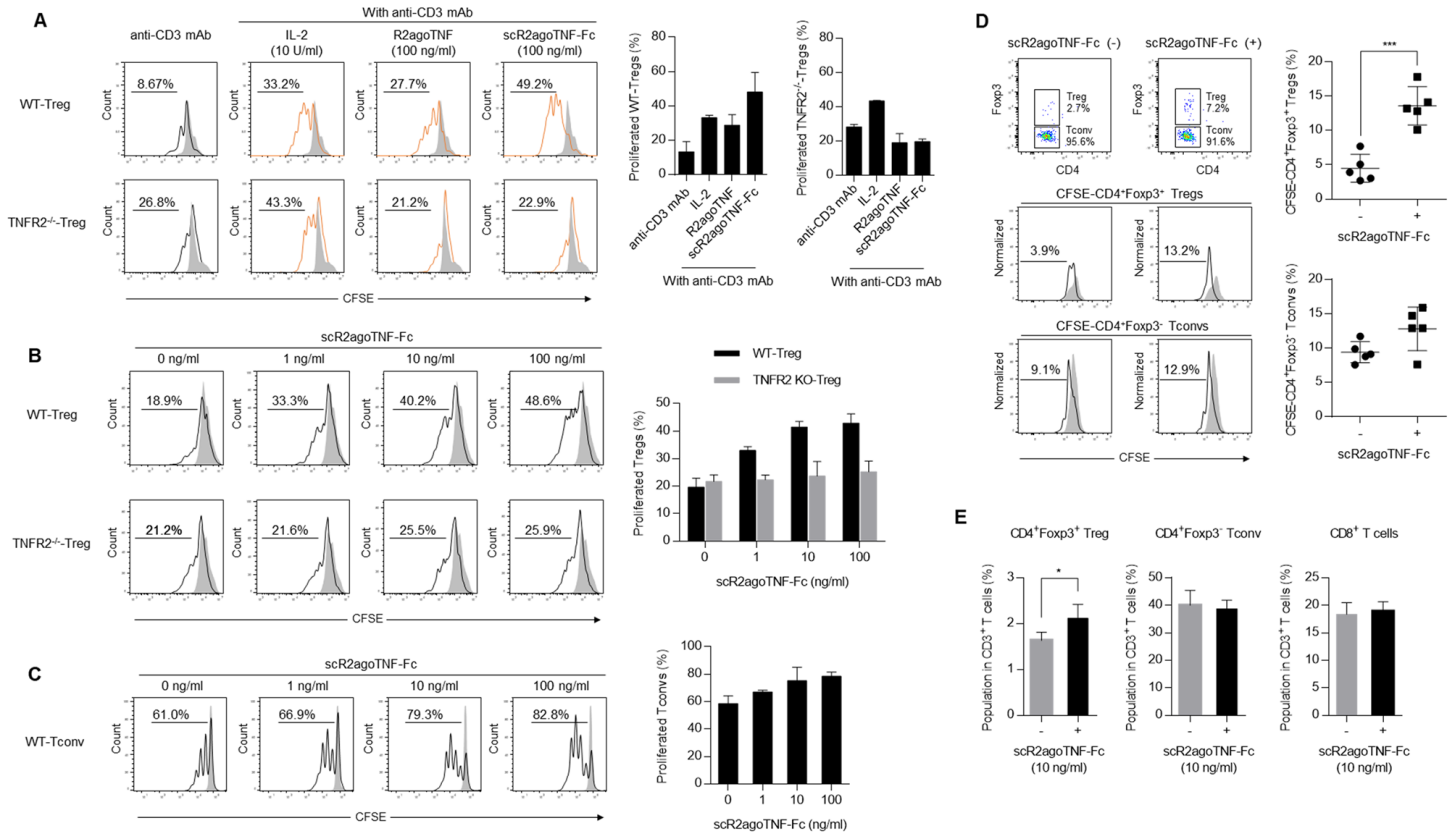
Ex vivo Treg expansion by scR2agoTNF-Fc. (**A**) Proliferation of CFSE-labeled WT-CD4^+^CD25^+^ Tregs or TNFR2^−/−^CD4^+^Foxp3^+^ Tregs isolated from WT-mouse LN or TNFR2^−/−^mouse LN, respectively. Each Treg population was stimulated for 72 h by IL-2 (10 U/ml), R2agoTNF-Fc (100 ng/ml), or scR2agoTNF-Fc (100 ng/ml) with immobilized anti-mouse CD3 mAb. Data are the mean ± SD (n = 4). Representative histograms are shown. (**B**) Concentration-dependency of scR2agoTNF-Fc and robustness of TNFR2 targeting for Treg expansion via TNFR2 were analyzed by a CFSE-labeled cell proliferation assay using WT-CD4^+^CD25^+^ Tregs and TNFR2^−/−^CD4^+^CD25^+^ Tregs. Data are the mean ± SD (n = 4). (**C**) Concentration-dependency of scR2agoTNF-Fc for CD4^+^CD25^−^ Tconvs expansion was analyzed. Data are the mean ± SD (n = 4). (**D**) Whole CD4^+^ T cells were immuno-stained with CFSE and cultured with scR2agoTNF-Fc (100 ng/ml) in immobilized anti-mouse CD3 mAb. Then, CFSE-positive cells were measured in CD4^+^Foxp3^+^ Tregs or CD4^+^Foxp3^−^ Tconvs to assess cell proliferation. Data are the mean ± SD (n = 5). ***p < 0.001 (unpaired Student’s *t*-test). (**E**) Populations of T cell subsets in mouse whole lymphocytes stimulated by scR2agoTNF-Fc were measured by FCM. Data are the indicated populations in CD3^+^ T cells (%). Data are the mean ± SD (n = 4). *p < 0.05 (unpaired Student’s *t*-test).

**Figure 3:**
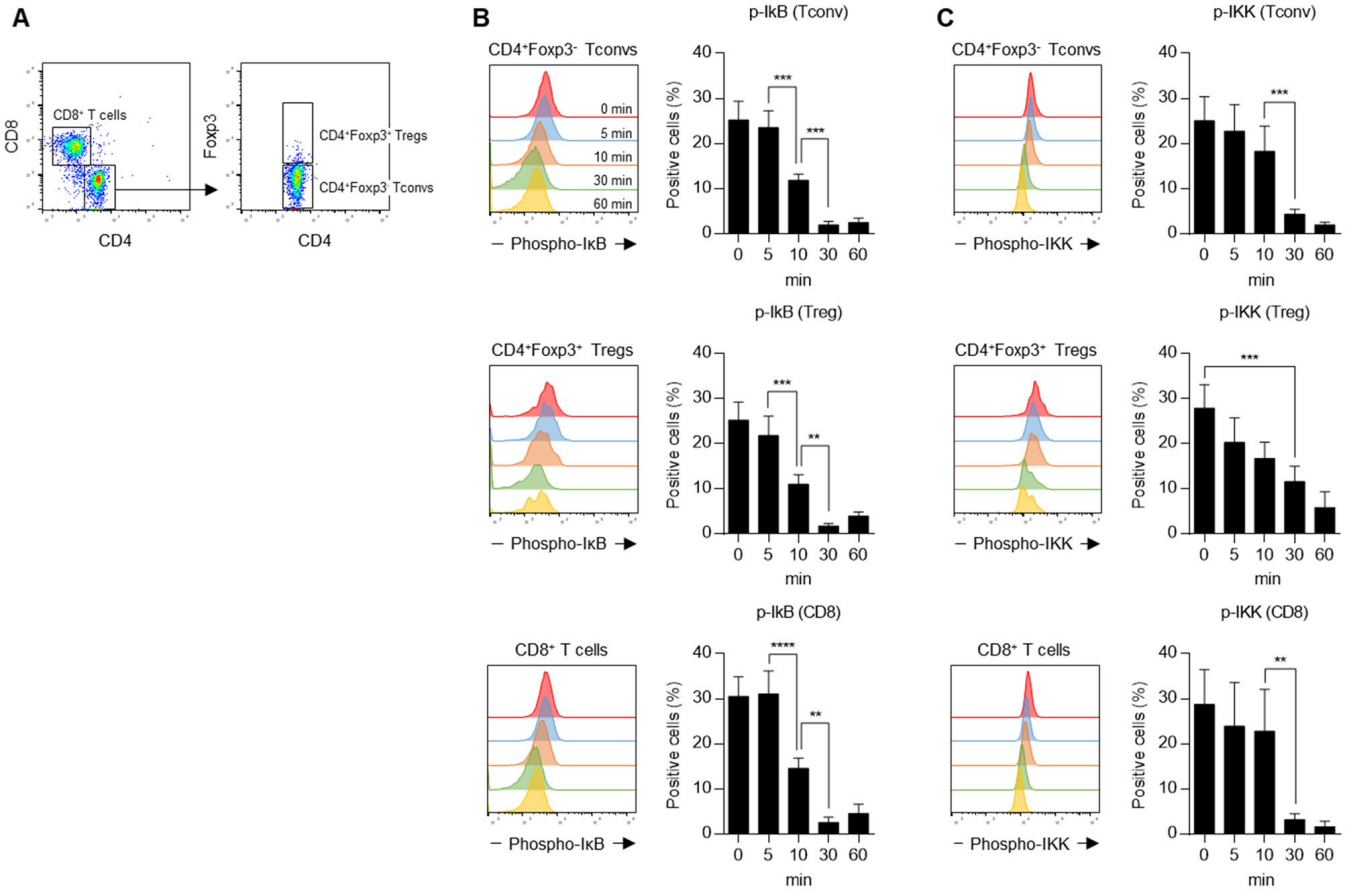
Canonical and non-canonical NF-κB signaling by the TNFR2 agonist-Fc. Intracellular NF-κB signaling in Tregs, Tconvs, and CD8^+^ T cells was assessed. (**A**) CD4^+^Foxp3^+^ Tregs, CD4^+^Foxp3^−^ Tconvs, and CD8^+^ T cells were gated in CD3^+^ T cells. (**B**) Each cell subset was stimulated by scR2agoTNF-Fc (100 ng/ml) for 0, 5, 10, 30, or 60 min. Then, to assess canonical NF-κB signaling activation, phospho-IκB positive cells in each subset were measured by the phosphoflow method. (**C**) To assess non-canonical NF-κB signaling activation, phospho-IKK positive cells in each subset were measured by the phosphoflow method. Data are the mean ± SD (n = 4). **p < 0.01, ***p < 0.001, ****p < 0.0001 (one-way ANOVA with Turkey multiple comparison test).

Treg proliferation activity of scR2agoTNF-Fc was measured ex vivo. CD4^+^CD25^+^ Tregs from mouse lymphocytes were labeled with CFSE and stimulated with IL-2, R2agoTNF, or scR2agoTNF-Fc with anti-mouse CD3 mAb. Proliferation rates of CD4^+^CD25^+^ Tregs were measured by CFSE division (Fig. 2A). scR2agoTNF-Fc enhanced Treg expansion compared with R2agoTNF. scR2agoTNF-Fc expanded WT-CD4^+^CD25^+^ Tregs, but not TNFR2^−/−^CD4^+^CD25^+^Tregs, by a concentration-dependent mechanism (Fig. 2B). Furthermore, scR2agoTNF-Fc expanded WT-CD4^+^CD25^−^ Tconvs under co-stimulation with immobilized anti-mouse CD3 mAb but showed a higher proliferative effect on Tregs (Fig. 2C). Thus, scR2agoTNF-Fc preferentially induced Treg proliferation ex vivo via TNFR2. Because these assays used CD4^+^CD25^+^ Tregs, it was not possible to confirm that the proliferating Tregs expressed Foxp3, which is the master transcription factor of Tregs. Therefore, another CFSE proliferation assay was performed with the co-culture of Tregs and Tconvs. CFSE-CD4^+^ cells were stimulated with scR2agoTNF-Fc for 72 h and then the number of CFSE divisions of CD4^+^Foxp3^+^ Tregs and CD4^+^ Foxp3^−^ Tconvs was measured. We found CFSE division of Tregs, but not Tconvs, was enhanced (Fig. 2D). Furthermore, we examined Treg preferential expansion by scR2agoTNF-Fc in co-cultures of Tregs and other T cell subsets. When whole lymph node cells were stimulated with scR2agoTNF-Fc for 72 h, CD4^+^Foxp3^+^ Tregs, but not CD4^+^Foxp3^−^ Tconvs and CD8^+^ T cells, were significantly increased (Fig. 2E). Additionally, intracellular NF-κB signaling in CD4^+^Foxp3^+^ Tregs, CD4^+^Foxp3^−^ Tconvs, or CD8^+^ T cells were assessed by phosphoflow using FCM (Fig. 3A). The phospho-IκB level in each cell decreased over time and was decreased significantly at 30 min (Fig. 3B). In addition, the phospho-IKK level in CD4^+^Foxp3^−^ Tconvs or CD8^+^ T cells was markedly decreased at 30 min similar to the phospho-IκB level, whereas the level in CD4^+^Foxp3^+^ Tregs decreased slowly (Fig. 3C).

### Enhancement of Treg suppression activity by scR2agoTNF-Fc

**Figure 4:**
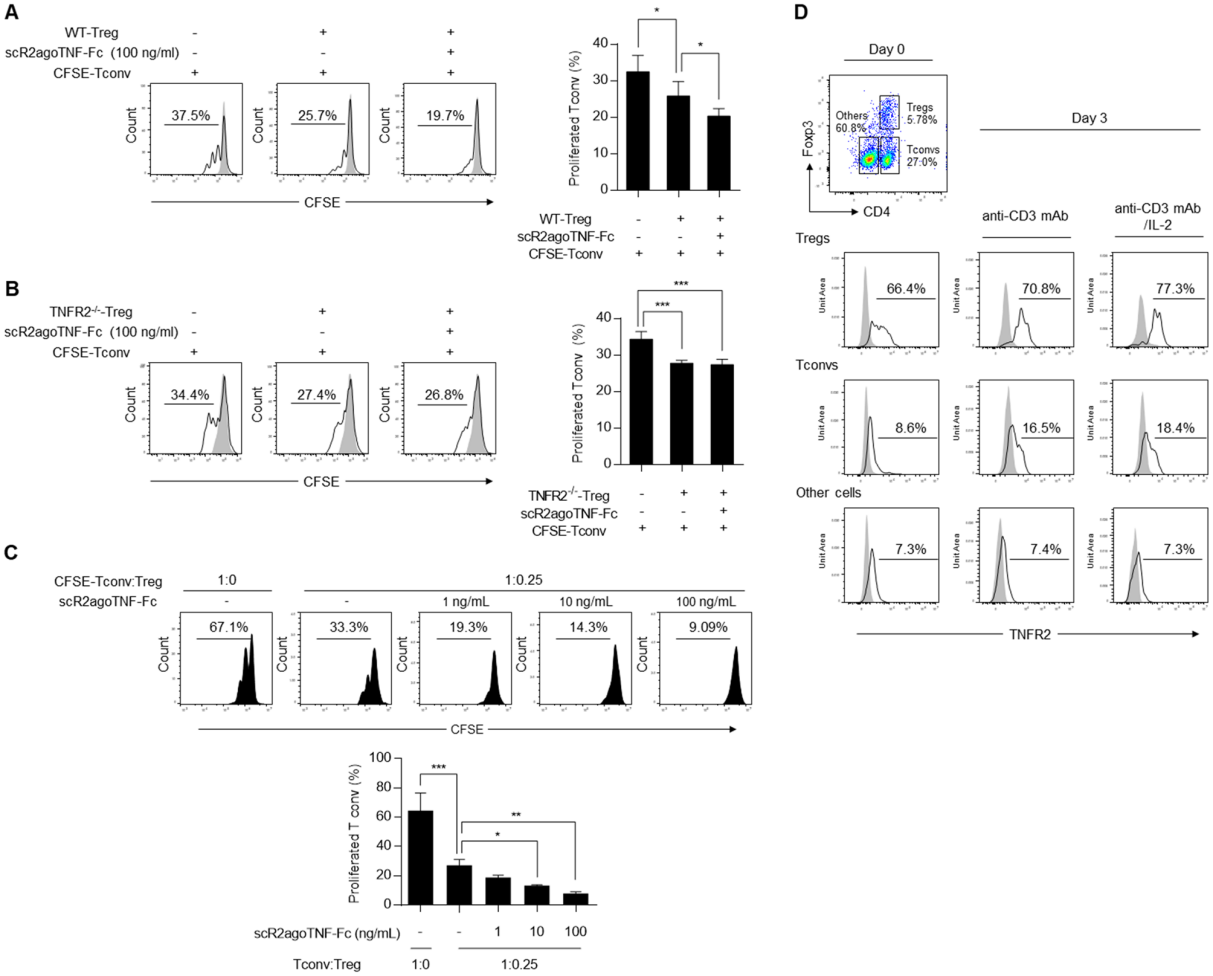
Ex vivo suppressive activity of Tregs expanded by scR2agoTNF-Fc. (**A**) WT-Tregs and (**B**) TNFR2^−/−^Treg suppression assays were performed using pre-expanded Tregs. WT-CD4^+^CD25^+^ Tregs and TNFR2^−/−^CD4^+^CD25^+^ Tregs were expanded by scR2agoTNF-Fc (100 ng/ml) for 72 h. Then, each Treg population was cultured with CFSE-labeled CD4^+^CD25^−^ Tconvs for another 72 h. CFSE division was measured by FCM. Data are the mean ± SD (n = 4). (**C**) A Treg suppression assay was performed under the conditions where CD4^+^CD25^+^ Tregs and CFSE-labeled CD4^+^CD25^−^ Tconvs were co-incubated. A mixture of Tregs, CFSE-Tconvs, and APC-containing 90.2^−^ cells were cultured and stimulated by scR2agoTNF-Fc (0, 1, 10, 100 ng/ml) for 72 h. Then, CFSE division was measured by FCM. Data are the mean ± SD (n = 4). (**D**) LN cells were stimulated with immobilized anti-mouse CD3 mAb and soluble recombinant IL-2 (10 U/ml). Then, TNFR2 expression levels of the activated T cell subsets, CD4^+^CD25^+^ Tregs, CD4^+^CD25^−^ Tconvs, and CD4^−^CD25^−^ cells, were measured. *p < 0.05, **p < 0.01, ***p < 0.001 (one-way ANOVA with Turkey multiple comparison test).

Tregs suppressed the proliferation of Tconvs. When CFSE-labeled CD4^+^CD25^−^ Tconvs were cultured with CD4^+^CD25^+^ Tregs, CFSE division was decreased dependent on Treg increments^12,21^. Therefore, although scR2agoTNF-Fc preferentially enhanced Treg expansion compared to Tconv, we examined whether Tregs expanded by scR2agoTNF-Fc suppressed Tconv proliferation. WT-CD4^+^CD25^+^ Tregs were expanded by stimulation from scR2agoTNF-Fc and immobilized anti-mouse CD3 mAb for 72 h and then co-cultured with CFSE-labeled CD4^+^CD25^−^ Tconvs for a further 72 h (Fig. 4A). CFSE division was decreased by co-cultivation with scR2agoTNF-Fc expanding WT-Tregs (19.7%). Statistical analysis indicated that WT-Tregs expanded by scR2agoTNF-Fc ex vivo significantly suppressed Tconv proliferation. However, TNFR2^−/−^Tregs stimulated by scR2agoTNF-Fc did not enhance Tconv suppression compared with unstimulated TNFR2^−/−^Tregs (Fig. 4B). Furthermore, we used a co-culture of Treg and Tconv to investigate whether Treg expansion by scR2agoTNF-Fc suppressed Tconv. When CFSE-labeled CD4^+^CD25^−^ Tconvs and CD4^+^CD25^+^ Tregs were stimulated with scR2agoTNF-Fc (Fig. 4C), the proliferation of CFSE-labeled Tconvs co-cultured with WT-Treg (Tconv:Treg = 1:0.25) (33.3%) was reduced compared with CFSE-labeled Tconvs alone (Tconv:Treg = 1:0) (67.1%). The addition of scR2agoTNF-Fc (1, 10, 100 ng/ml) further reduced the proliferation of CFSE-Tconvs by a concentration-dependent mechanism. scR2agoTNF-Fc enhanced the suppression of Tconv proliferation by Tregs even when Tconvs and Tregs were co-incubated. scR2agoTNF-Fc expanded and activated Tregs via TNFR2, which is preferentially expressed by Tregs compared with other lymphocyte subsets^12^. Therefore, TNFR2 might be a potential target for Treg expansion. To evaluate the robustness of TNFR2 expression levels, changes in TNFR2 expression levels on activated CD4^+^Foxp3^+^ Tregs, CD4^+^Foxp3^−^ Tconvs, and CD4^−^Foxp3^−^ cells were estimated (Fig. 4D). TNFR2 expression levels of Tregs were retained by stimulation with anti-mouse CD3 mAb or recombinant IL-2 whereas the TNFR2 levels on Tconvs or other cells remained low suggesting TNFR2 activation by scR2agoTNF-Fc preferentially expanded Tregs.

### Plasma clearance and Treg expansion activity of scR2agoTNF-Fc in vivo

**Figure 5:**
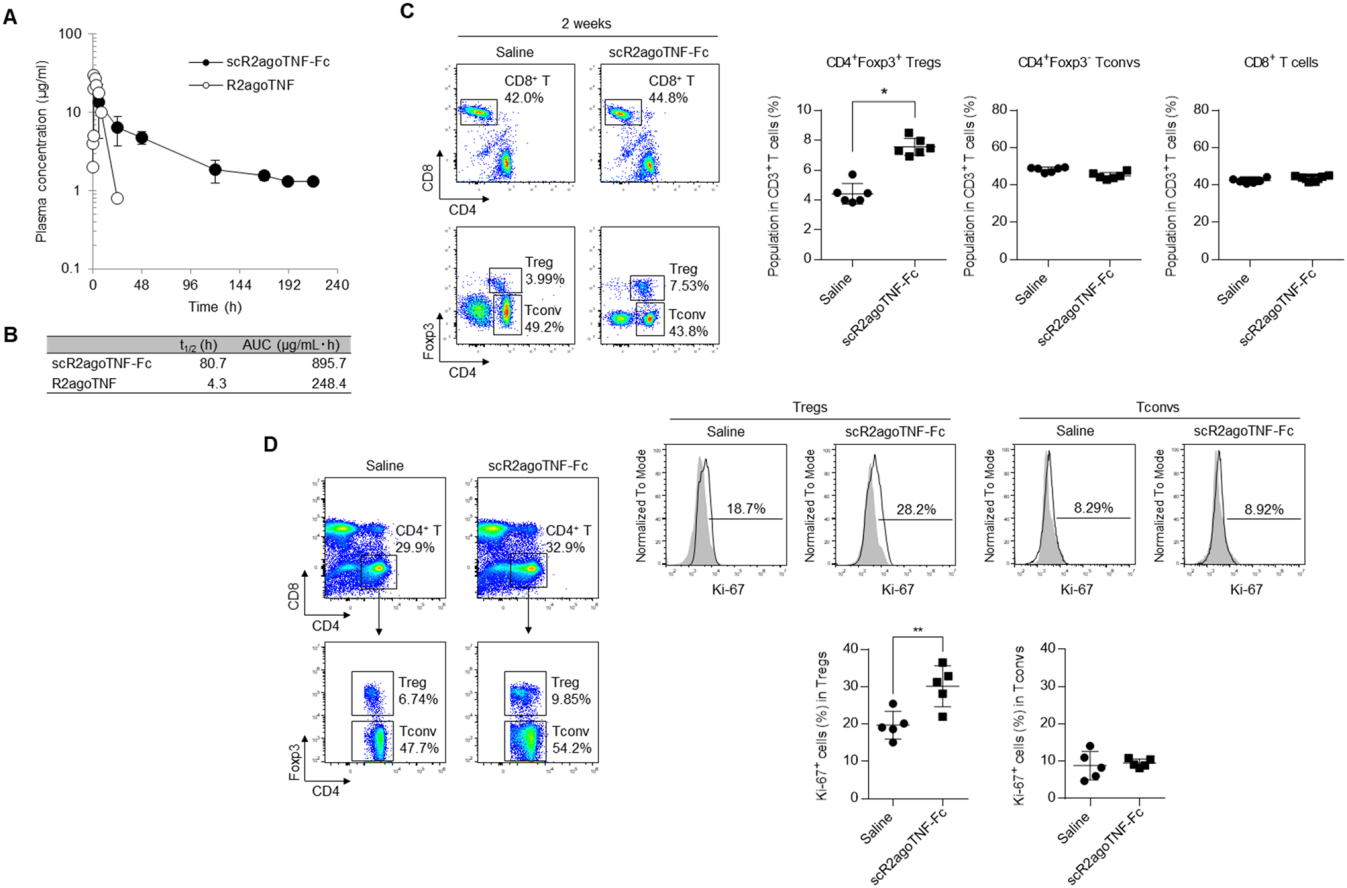
In vivo plasma clearance and Treg expansion activity of scR2agoTNF-Fc. (**A**) Plasma clearances of scR2agoTNF-Fc and R2agoTNF (50 μg/mouse each) were confirmed after a single i.p. injection. Plasma concentrations were measured by ELISA for human TNF-α or human IgG-Fc. Data are the mean ± SD (n = 6). (**B**) The t½ and AUC were calculated from time-concentration curves by moment analysis. Data are the mean ± SD (n = 6). (**C**) Populations of CD4^+^CD25^+^ Tregs, CD4^+^CD25^−^ Tconvs, and CD8^+^ T cells in CD3^+^ T cells were assessed after the administration of scR2agoTNF-Fc (50 μg/mouse i.p., twice a week) for 2 weeks. Each population was gated separately due to differences in the cell distribution between scR2agoTNF-Fc-administration and saline-administration groups. Data are the mean ± SD (n = 5). *p < 0.05 (unpaired Student’s *t*-test). (**D**) After 2 weeks administration of scR2agoTNF-Fc (50 μg/mouse i.p., twice a week) or saline, populations of Ki-67^+^ cells in CD4^+^Foxp3^+^ Tregs or CD4^+^Foxp3^−^ Tconvs were measured. Data are the mean ± SD (n = 6). **p < 0.01 (unpaired Student’s *t*-test).

We previously reported that scR2agoTNF-Fc expanded Tregs in vivo^21^. Therefore, to evaluate the retention of scR2agoTNF-Fc in vivo, plasma concentrations of scR2agoTNF-Fc were measured after a single intraperitoneal (i.p.) administration to mice (Fig. 5A). R2agoTNF, an Fc un-fusion protein, disappeared from the blood within 24 h after administration with a half-life (t½) of 4.3 h (Fig. 5B). By contrast, plasma concentrations of scR2agoTNF-Fc peaked 90 min after i.p. administration and were maintained for 4 days. The t½ of scR2agoTNF-Fc was 80.7 h, which was extended compared with R2agoTNF. The area under the curve (AUC) of scR2agoTNF-Fc was 3.5-fold greater than that of R2agoTNF. R2agoTNF required i.p. administration twice a day to maintain a concentration in blood (data not shown). However, scR2agoTNF-Fc was expected to reduce the frequency of administration, because the Fc fusion extended its half-life in blood. To validate the Treg expansion effect of scR2agoTNF-Fc in vivo, we administered scR2agoTNF-Fc to mice twice a week, then measured cell populations of T cell subsets in the lymph nodes (LN) after 2 weeks (Fig. 5C). CD4^+^CD25^+^ Tregs were significantly increased in the scR2agoTNF-Fc-injection group compared with the saline-injection group. By contrast, populations of CD4^+^CD25^−^ Tconvs and CD8^+^ T cells were not significantly changed by the scR2agoTNF-Fc injection. Additionally, in a separate experiment, a population of Ki-67^+^ cells in CD4^+^CD25^+^ Tregs was also increased by scR2agoTNF-Fc compared with saline (Fig. 5D). However, the Ki-67^+^ population in CD4^+^CD25^−^ Tconvs was not increased by scR2agoTNF-Fc. Therefore, scR2agoTNF-Fc preferentially expanded Tregs in vivo.

### Effects of scR2agoTNF-Fc on the fluorescein isothiocyanate (FITC)-induced CHS model mice

**Figure 6:**
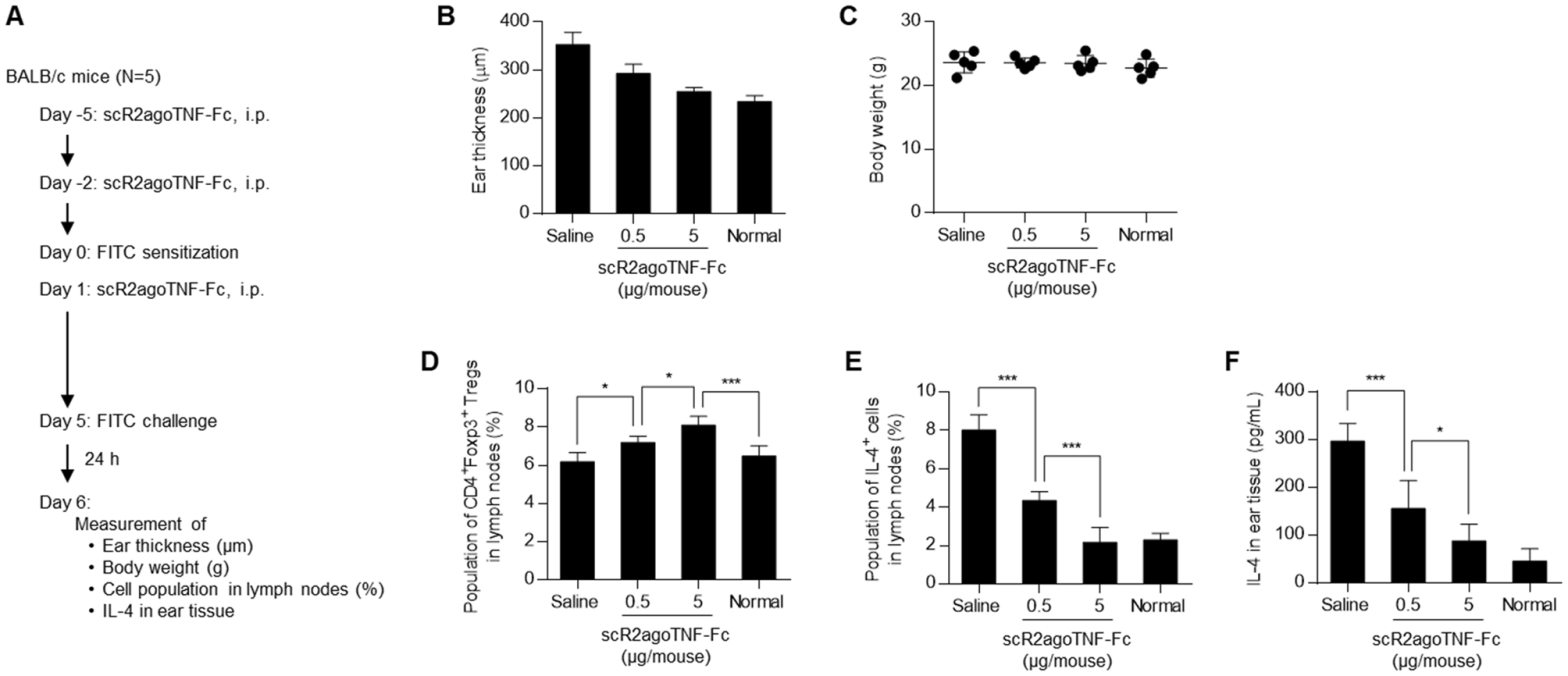
Effects of scR2agoTNF-Fc on FITC-sensitized contact hypersensitivity mice. (**A**) BALB/c mice were sensitized with 0.5% FITC painting of the abdominal skin (n = 5). scR2agoTNF-Fc (0.5 and 5 μg/mouse i.p., respectively) was prophylactically administered before FITC challenge as described in the schedule chart. (**B**) Swelling measured by ear thickness and (**C**) bodyweights of each group were assessed. (**D**) Populations of CD4^+^Foxp3^+^ Tregs and (**E**) IL-4^+^ cells in regional LN were measured 24 h after FITC challenge. (**F**) Concentration of mouse IL-4 in plasma 24 h after FITC challenge was measured by ELISA. Data are the mean ± SD (n = 5). *p < 0.05, ***p < 0.001 (one-way ANOVA with Turkey multiple comparison test).

2,4-Dinitrofluorobenzene induced type I helper T cell (Th1)-driven CHS in BALB/c mice^25^. We previously reported that scR2agoTNF-Fc suppressed Th1-driven CHS conditions including ear swelling and IFN-γ production^21^. By contrast, FITC induced Th2-driven CHS related to IL-4 production^26,27^. In this study, we confirmed the effect of scR2agoTNF-Fc on FITC-induced CHS model mice. Prior to the first immunization with FITC, scR2agoTNF-Fc was pre-administered to mice twice every 3 days (Fig. 6A). Five days after the first immunization, FITC was re-applied to ear surfaces and inflammation was measured 24 h later. Ear thickness was suppressed dose-dependently in the scR2agoTNF-Fc group (Fig. 6B). Loss of bodyweight was not promoted by scR2agoTNF-Fc treatment (Fig. 6C) and no adverse effects were noted. CD4^+^Foxp3^+^ T cells in the regional LN were increased dose-dependently by scR2agoTNF-Fc (Fig. 6D). IL-4 production by effector T cells in the regional LN was decreased in scR2agoTNF-Fc-treated mice compared with saline-treated mice (Fig. 6E). IL-4 concentrations in ear tissues were dose-dependently decreased by scR2agoTNF-Fc (Fig. 6F). Thus, scR2agoTNF-Fc is effective for Th2-type CHS.

### Effects of scR2agoTNF-Fc in collagen-induced arthritis (CIA) model mice

**Figure 7:**
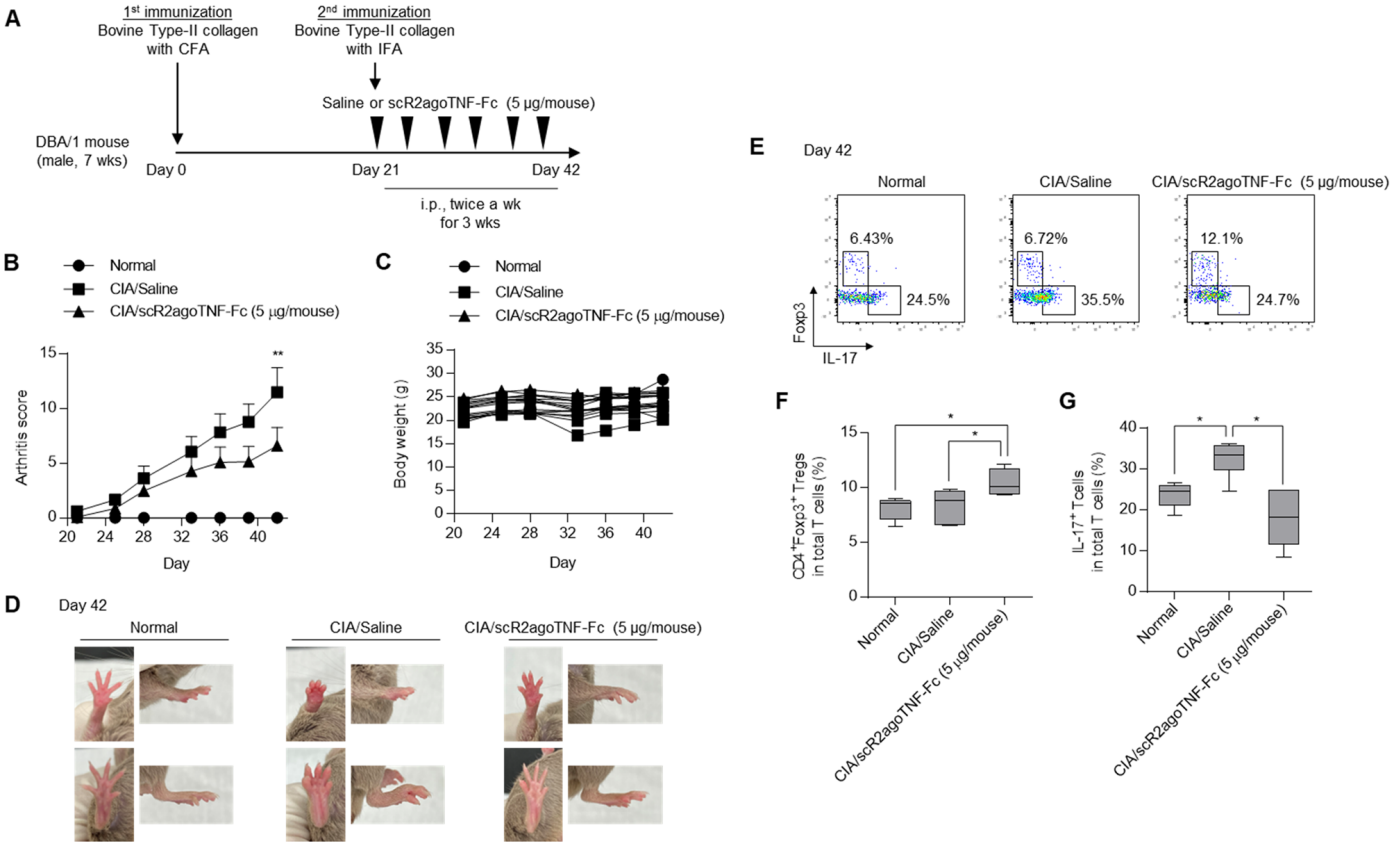
Effects of scR2agoTNF-Fc on CIA mice. (**A**) DBA/1 mice were immunized by the subcutaneous tail injection of bovine type II collagen in CFA (1st immunization) followed by immunization with type II collagen in IFA at day 21 (2nd immunization). Saline (n = 6) and scR2agoTNF-Fc (5 μg/mouse) (n = 6) were administered i.p. twice a week from day 21. (**B**) The sum of arthritis scores of four paws and (**C**) bodyweight were measured for 3 weeks. **p < 0.01 (one-way ANOVA with Turkey multiple comparison test). (**D**) Joint swelling of front and hind limbs from a representative mouse from each group at day 42 is shown. (**E**) Populations of CD4^+^Foxp3^+^ Tregs and CD4^+^IL-17^+^ cells in mouse LN administered saline or scR2agoTNF-Fc (5 μg/mouse), respectively, at day 42 were measured by FCM. Representative data are shown as a dot plot. Statical analysis of (**F**) CD4^+^Foxp3^+^ Tregs and (**G**) CD4^+^IL-17^+^ cells in CD3^+^ T cells are indicated. Data are the mean ± SD (n = 6). *p < 0.05 (one-way ANOVA with Turkey multiple comparison test).

CIA is a widely-used chronic autoimmune model of human RA used to evaluate potential therapeutic drugs^28^. To evaluate whether scR2agoTNF-Fc suppressed inflammation related to arthritis, scR2agoTNF-Fc was administered to type II CIA mice twice a week for 3 weeks (Fig. 7A). The degree of arthritis was significantly decreased in scR2agoTNF-Fc (25 μg/kg)-treated mice compared with saline-treated mice (Fig. 7B). The bodyweight of scR2agoTNF-Fc-treated mice did not decrease compared with saline-treated mice (Fig. 7C). At 42 days after dosing for 3 weeks, severe limb swelling was observed in saline-treated mice compared with normal mice. By contrast, swelling in scR2agoTNF-Fc (25 μg/kg)-treated mice was suppressed (Fig. 7D). To confirm the expansion of Tregs in the regional LN by scR2agoTNF-Fc, we measured CD4^+^Foxp3^+^ Tregs in total T cells (Fig. 7E). Statistical analysis indicated that Tregs were significantly increased in scR2agoTNF-Fc-treated mice compared with saline-treated mice (Fig. 7F). Furthermore, T cells positive for IL-17, an inflammatory cytokine, were decreased by scR2agoTNF-Fc (Fig. 7G), suggesting joint swelling caused by inflammatory cytokines was suppressed by Treg expansion induced by scR2agoTNF-Fc.

### Treg expansion activity of a human TNFR2 agonist

**Figure 8:**
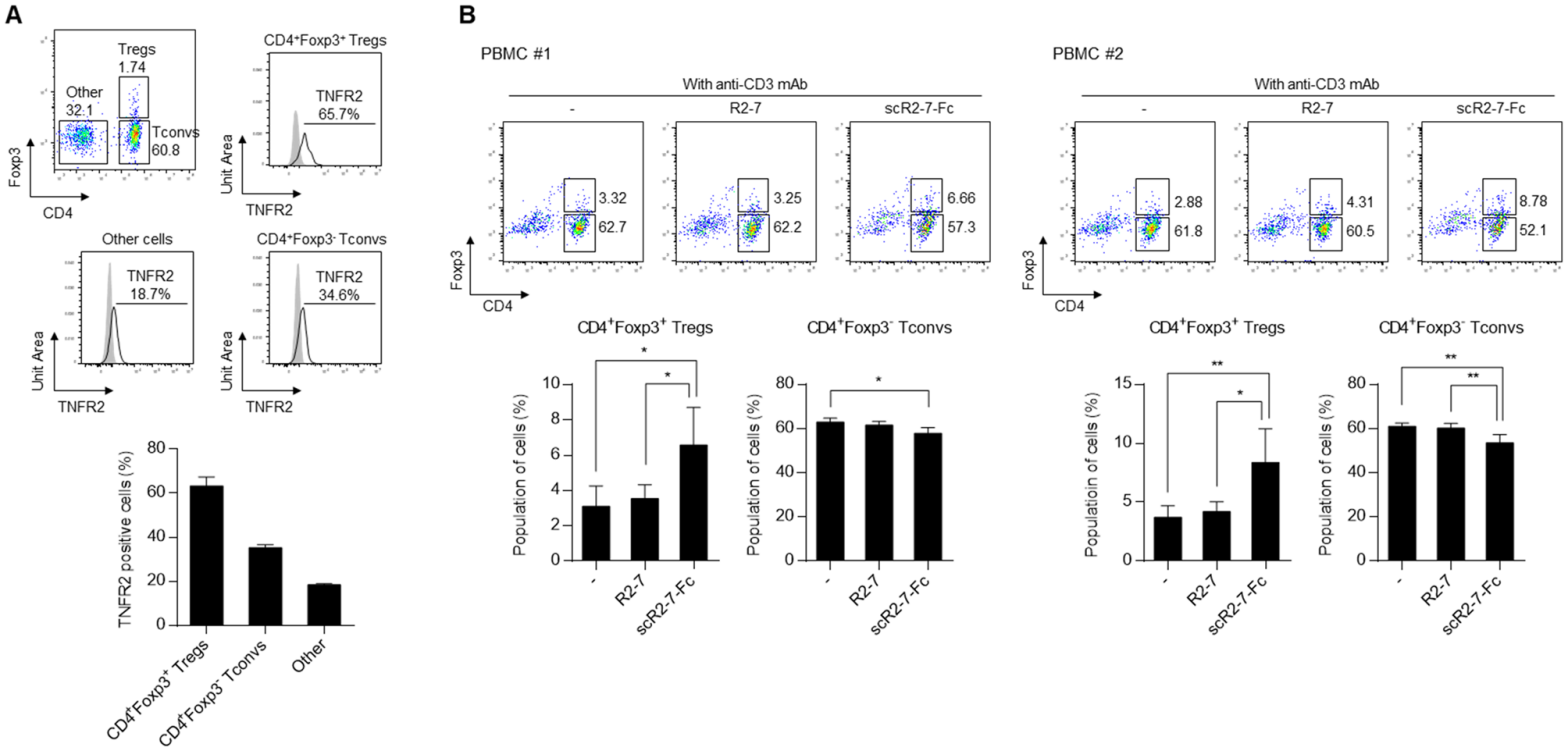
Treg expansion activity by a human TNFR2 agonist. (**A**) Human TNFR2 expression levels on T cell subsets, CD4^+^Foxp3^+^ Tregs, CD4^+^Foxp3^−^ Tconvs, and CD4^−^Foxp3^−^ cells, from human PBMCs. Populations of TNFR2 positive cells in each cell subset were measured by FCM. Data are the mean ± SD (n = 3). (**B**) Populations of CD4^+^Foxp3^+^ Tregs and CD4^+^Foxp3^−^ Tconvs were compared after the stimulation of human PBMCs by R2-7 (100 ng/ml) or scR2-7-Fc (100 ng/ml) for 72 h. PBMC #1 and #2 were obtained from different donors. Data are the mean ± SD (n = 5). *p < 0.05, **p < 0.01 (one-way ANOVA with Turkey multiple comparison test).

scR2agoTNF-Fc selectively binds to mouse TNFR2, not human TNFR2. We previously generated a human TNF-α mutated protein, R2-7, which binds to human TNFR2^19^. Here, we created a single-chain R2-7 IgG-Fc fusion protein, scR2-7-Fc, that targeted human TNFR2. scR2agoTNF-Fc is a mouse TNFR2-targeted surrogate agonist of scR2-7-Fc. Human Tregs express TNFR2 on their cell surface^22,29^. We confirmed TNFR2 expression levels on CD4^+^Foxp3^+^ Tregs, CD4^+^Foxp3^−^ Tconvs, and other cells using human peripheral blood mononuclear cells (PBMCs). FCM showed that Tregs preferentially expressed TNFR2 compared with Tconvs or other cells (Fig. 8A). To evaluate human Treg expansion by TNFR2 agonists, PBMCs from two donors (PBMC #1 and #2) were cultured with anti-human CD3 mAb, R2-7, or scR2-7-Fc. R2-7 did not markedly induce Treg proliferation activity in PBMC #1 (Fig. 8B, left). However, scR2-7-Fc significantly expanded CD4^+^Foxp3^+^ Tregs compared with other groups. Similar expansion profiles were observed in PBMC #2 (Fig. 8B, right). These results indicated that scR2-7-Fc with the bivalent structure of a TNFR2 agonist based on Fc fusion, was effective for preferential Treg expansion via TNFR2.

## Discussion

Tregs are essential for maintaining self-tolerance and immune cell homeostasis. They regulate immune responses during autoimmune diseases, organ transplantation rejection, or GvHD^30–32^. Therefore, these diseases might be suppressed by modulating the number and activity of Tregs. Although Tregs are potent suppressors of other immune cell subsets, they are not abundant. For example, Treg expansion in vivo by low-dose IL-2 was attempted to acquire immune tolerance for the treatment of GvHD^33,34^. In addition, Treg adoptive transfer to humans was investigated for allograft rejection after liver transplantation^32,35^. Furthermore, the Treg inducing molecule, AS2863619, a cyclin-dependent kinase 8 (CDK8) and CDK19 inhibitor, converted antigen-specific effector/memory T cells into Foxp3^+^ Treg cells for the treatment of various immunological diseases^36^. Thus, Tregs might be a promising target for immune tolerance, and new molecules that induce Treg proliferation/activation are being explored. We created a new modality, TNFR2-selective agonistic TNF-α mutated proteins, as Treg expanders. Here, we investigated a Treg expansion methodology ex vivo and in vivo using a TNFR2 agonist, scR2agoTNF-Fc.

IL-2 was reported to expand Tregs through CD25, a high-affinity IL-2 receptor^37^. Low-dose IL-2 is used clinically to suppress immune responses in GvHD patients^34,38,39^; however, high-dose IL-2 enhances the risk of dose-dependent adverse effects by activating effector T cells. Recently, TNFR super families including GITR, OX-40, FR-4, and especially TNFR2 (highly and preferentially expressed on Tregs in mice and humans) were considered alternative target molecules of CD25 to expand Tregs^11,40–42^. TNF-α, an endogenous ligand for TNFR, expanded Tregs in vitro via TNFR2^11^. However, the administration of TNF-α in vivo induces serious adverse effects including liver failure, hepatic failure, and systemic shock related to TNFR1 agonism^43^. However, TNFR2 agonism suppressed RA, GvHD, or T1D in mouse models or human subjects via Treg expansion/activation. For example, STAR2, a mouse TNF-based TNFR2 agonist, prolonged the survival and decreased the severity of disease in mouse models of GvHD^44^ and RA^45^. Our human TNF-based mouse TNFR2 agonist, R2agoTNF, specifically transmits signaling via mouse TNFR2^20,21^. We investigated whether R2agoTNF expanded Tregs ex vivo and in vivo. Ex vivo cell proliferation assays demonstrated that IL-2 expanded CD4^+^CD25^+^ Tregs and CD4^+^CD25^−^ Tconvs. In contrast, R2agoTNF preferentially expanded CD4^+^CD25^+^ Tregs with the upregulated expression of CD25 and CTLA-4, and increased the production of IL-10 and TGF-β. R2agoTNF and recombinant TNF-α similarly expanded Tregs ex vivo (Supplementary Fig. S2). However, R2agoTNF has poor in vivo stability and a half-life in blood < 24 h, because it is a low molecular-weight soluble protein derived from human TNF-α. Furthermore, R2agoTNF administered i.p. barely expanded CD4^+^Foxp3^+^ Tregs in mice. Therefore, we optimized the R2agoTNF structure by IgG-Fc fusion to enhance its agonistic activity and molecular stability. We found enhanced TNFR2 signaling by oligomerization of FLAG-tagged TNFR2 agonist. In a cytotoxicity assay in which TNFR2/Fas-expressing cells were treated with a TNF-α or a R2-7 with a FLAG-tag fused to the C-terminus (TNF-FLAG or R2-7-FLAG, respectively), adding the anti-FLAG-tag mAb enhanced cytotoxicity (Supplementary Fig. S3A and S3B). The result demonstrated that TNFR2 agonist activity was enhanced by dimerization or multimerization. Furthermore, we previously reported a TNFR1-selective antagonist TNF-α mutein Fc fusion protein, scR1antTNF-Fc, with a prolonged half-life in blood^24,46^. The antagonist protein suppressed inflammation similar to Enbrel, a clinically-used TNF inhibitor, even at low doses in RA mice. Here, we generated a new modality Treg expander, scR2agoTNF-Fc, based on R2agoTNF, using our methodology with combined single-chain and Fc-fusion. scR2agoTNF-Fc can be expressed from a single plasmid in mammalian cells into which the R2agoTNF and Fc fusion gene is inserted (Fig. 1A,B). Furthermore, proteins can be easily purified by affinity chromatography for Fc. These are advantages for expressing and isolating proteins. scR2agoTNF-Fc was produced using a mammalian cell expression system and was successfully obtained as a fusion protein of the expected molecular size without aggregation. Results from BIAcore showed that scR2agoTNF-Fc, even by Fc fusion, exhibited TNFR2 selectivity, binding to TNFR2 but not to TNFR1, similar to R2agoTNF (Fig. 1E,F).

TNF-α has two molecular forms, soluble TNF and membrane-bound TNF. The oligomerization of TNFR2 by membrane-bound TNF fully activates its intracellular signaling^23,47^. We previously found that TNF-TNFR2 aggregates on the cell surface were required for signal initiation^48^. Because scR2agoTNF-Fc contains a bivalent R2agoTNF structure by the IgG-Fc fusion, the protein forms a ligand-TNFR2 cluster. Therefore, scR2agoTNF-Fc might enhance TNFR2 signaling similar to membrane-bound TNF by forming a TNFR2 agonist-TNFR2 cluster. In undenatured western blotting, the molecular size of a mixture of scR2agoTNF-Fc and recombinant TNFR2 protein was larger than the expected molecular size of the 1:1 binding of scR2agoTNF-Fc and TNFR2 (Fig. 1H). This suggested that scR2agoTNF-Fc formed a TNFR2 cluster. Compared with R2agoTNF, scR2agoTNF-Fc enhanced cell-sorted CD4^+^CD25^+^ Treg proliferation via TNFR2 ex vivo (Fig. 2A). A recombinant IL-2 enhanced the proliferation of Tregs ex vivo^21^. However, we found that CD4^+^CD25^+^ Tregs were proliferated by immobilized anti-mouse CD3 mAb or by a combination of immobilized anti-mouse CD3 mAb and our TNFR2 agonist. In a previous report^21^, we confirmed that anti-IL-2 mAb suppressed IL-2-induced Treg proliferation, but not scR2agoTNF-Fc-induced Treg proliferation. Most of the isolated CD4^+^CD25^+^ Tregs (> 90%) were Foxp3^+^ cells, suggesting that there was little contamination of Tconvs (Supplementary Fig. S4). Therefore, even in the absence or less of IL-2, the activation of TNFR2 signaling was thought to enhance Treg proliferation. Pre-expanded CD4^+^CD25^+^ Tregs had a sustained suppressive activity on CD4^+^CD25^−^ Tconv proliferation (Fig. 4A). scR2agoTNF-Fc does not directly induce Tconv cell death. Moreover, without recombinant IL-2, scR2agoTNF-Fc preferentially enhances Tregs proliferation rather than Tconvs proliferation. However, in a suppression assay using Tregs co-cultured with Tconvs, it is possible that IL-2 released from Tconvs stimulates Tregs. In either situation, Tregs expanded by scR2agoTNF-Fc ex vivo certainly exhibit immunosuppressive activity, because scR2agoTNF-Fc acts additively with IL-2. In addition, when scR2agoTNF-Fc stimulated CD4^+^ T cells, the CD4^+^Foxp3^+^ Treg subset was significantly increased compared with the CD4^+^Foxp3^−^ Tconv subset. These proliferation assay results supported the idea that scR2agoTNF-Fc suppresses Tconv proliferation in co-cultures of CD4^+^CD25^+^ Tregs and CD4^+^CD25^−^ Tconvs (Fig. 4C). Therefore, scR2agoTNF-Fc can expand Tregs ex vivo, and might be useful for adoptive cell transfer therapy.

NF-κB has essential roles in multiple physiological functions via the canonical and non-canonical NF-κB pathways. The canonical NF-κB pathway is activated to respond to diverse external stimuli involved in inflammation, immune responses, and cell proliferation, differentiation, and survival^49,50^. Activation of the canonical NF-κB pathway is evoked by the phosphorylation-dependent activation of the IKKs (IκB kinases) complex, and subsequent ubiquitination-dependent degradation by proteasomes induced by phosphorylated inhibitory IκB proteins. In contrast, the non-canonical NF-κB is activated primarily via TNF superfamily receptors, indicating that the biological functions of this pathway are more limited. NIK (NF-κB inducing kinase) phosphorylates the IKK complex, which phosphorylates p100 (IκB domain) of the p100/RelB complex. TNFR2 binding to cell membrane-bound TNF activates the NF-κB-induced non-canonical pathway^51,52^. Phosphoflow analysis confirmed that stimulation of scR2agoTNF-Fc might activate non-canonical NF-κB signaling pathways in Tregs to enhance their proliferation or immunosuppressive functions, because CD4^+^Foxp3^+^ Tregs stimulated by scR2agoTNF-Fc supported a phosphorylation of IKKα compared with that of IkB (Fig. 3B,C). However, NIK transgenic-Treg had been reported to reduce the immunosuppressive activity of Treg ex vivo^53^. Therefore, excessive NIK activity seemed not to be required for Treg activation, and even p-IKKα level in Tregs was decreased relatively quickly.

Fc-fusion increased the molecular stability in vivo (Fig. 5A,B) and scR2agoTNF-Fc preferentially expanded CD4^+^Foxp3^+^ Tregs compared with CD4^+^ Foxp3^−^ Tconvs or CD8^+^ T cells in mice in vivo (Fig. 5C). Tregs in the LN of scR2agoTNF-Fc treated mice had a higher expression of Ki-67, a marker of proliferation, than those of saline-treated mice (Fig. 5D). Therefore, scR2agoTNF-Fc contributed to Treg expansion directly in vivo with a lower dose frequency because the Fc-fusion allowed for longer dosing intervals. scR2agoTNF-Fc expand Tregs directly in vivo. Skin CHS reactions are a type IV allergy response with a delayed hypersensitivity reaction. FITC-induced hypersensitivity is evoked by CD4^+^ Th2 lymphocytes expressing IL-4, a Th2 cytokine^25^. Administration of scR2agoTNF-Fc prior to FITC challenge suppressed ear inflammation induced by FITC (Fig. 6B) and there was a relationship between decreased IL-4^+^ T cells and increased Tregs (Fig. 6D,E). This might explain the anti-inflammatory mechanism of scR2agoTNF-Fc in type IV allergy. CIA evoked severe inflammation of mouse limbs. In our study, inflammation was initiated by secondary collagen immunization. However, administration of scR2agoTNF-Fc after secondary immunization suppressed inflammation with increased Tregs and decreased IL-17^+^ T cells (Fig. 7). These findings suggest scR2agoTNF-Fc might have potential therapeutic effects on autoimmune diseases via Treg expansion in vivo.

scR2agoTNF-Fc is a useful analytical tool as a surrogate agonist to verify the function of mouse Tregs. We generated a TNFR2 agonistic TNF-α mutein for human TNFR2, scR2-7-Fc, to verify TNFR2 agonism for immunosuppressive activity via human Tregs^19^. Several anti-human TNFR2 monoclonal antibodies that expand Tregs have been reported^22,29,54^. scR2-7-Fc is also expected to expand human Tregs via TNFR2 signaling. The CD4^+^Foxp3^+^ Treg fraction of PBMCs obtained from two human donors was preferentially expanded by scR2-7-Fc, compared with CD4^+^ Tconvs or CD8^+^ T cells. Therefore, scR2-7-Fc is expected to induce Treg proliferation in humans in vivo.

Here, we generated novel Treg expanders based on human TNF-α and showed they preferentially expanded Tregs ex vivo and in vivo*.* These TNFR2 agonists have improved molecular properties associated with Fc fusion including (i) increased avidity for TNFR2 related to the bivalent agonist structure, (ii) extended retention in blood (improved pharmacokinetics) due to an increase in its molecular weight, and (iii) enhanced signaling activity associated with its receptor clustering effect. Structure optimization was achieved to effectively expand and activate Tregs. scR2agoTNF-Fc might be a powerful tool to examine Treg functions by targeting mouse TNFR2, and scR2-7-Fc might be a potential clinical drug.

## Methods

### Mice

C57BL/6 WT mice were purchased from Oriental Yeast Co., Ltd. (Tokyo, Japan). B6.129S2-Tnfrsf1b^tm1Mwm^/J (TNFR2^−/−^) mice were purchased from The Jackson Laboratory (Bar Harbor, ME) and bred under specific pathogen-free conditions. All animal studies were approved by the Kobe Gakuin University Experimental Animal Care and Use Committee (approval number A22-18 and A22-19; Kobe, Japan). All animal experiments were performed in compliance with the ARRIVE guidelines.

### Single-cell preparation from lymphoid tissues

Mice were sacrificed by an overdose administration (i.p.) of sodium pentobarbital. Single-cell suspensions from mesenteric, inguinal, axillary LN, or spleens of mice were prepared by filtration through a 70-μm cell strainer (BD Biosciences, San Jose, CA). Erythrocytes were lysed using BD Pharm Lyse (BD Biosciences) according to the manufacturer’s protocol. Freshly prepared cells were used in each experiment.

### Cell culture

LN cells or splenocytes were cultured with RPMI-1640 (FUJIFILM Wako Pure Chemical Corporation, Osaka, Japan) supplemented with 10% fetal bovine serum (FBS) (Biowest, Nuaillé, France), 1% antibiotic mixture (10,000 U/ml penicillin, 10 mg/ml streptomycin, 25 μg/ml amphotericin B; FUJIFILM Wako), 50 μM 2-mercaptoethanol (FUJIFILM Wako), and 1% Non-essential Amino Acid Solution (FUJIFILM Wako). Mouse TNFR2/Fas preadipocytes (TNFR1^−/−^/TNFR2^−/−^ mouse preadipocytes expressing the chimeric receptor fused extracellular and transmembrane domains of mouse TNFR2 and intracellular domain of mouse Fas) were previously established^55^ and cultured in Dulbecco’s modified Eagle’s medium supplemented with 10% FBS, 1% antibiotic mixture, and 5 μg/ml blasticidin S HCl (all FUJIFILM Wako).

### Structural modeling of the TNFR2 agonist-Fc

The monomeric scR2agoTNF-Fc structure was modeled using the I-TASSER Protein Structure and Function Predictions program (https://zhanggroup.org/I-TASSER/). The dimeric structure was constructed to superimpose that of human IgG1 Fc (PDB code 1E4K).

### Cloning of the scR2agoTNF-Fc gene

The gene sequence of R2agoTNF was reported previously^20^. The scR2agoTNF gene (GenScript, Piscataway, NJ, USA) was fused to a signal peptide gene derived from the human IgG Vh region (MGWSLILLFLVAVATGVHS) on the N-terminal by PCR. The resultant gene was inserted into a pCAG-Hyg human IgG-Fc fusion vector (Fujifilm Wako) to generate the scR2agoTNF-Fc expression vector.

### Expression and purification of TNF-α mutein

scR2agoTNF-Fc protein was expressed using the Expi293 Expression System (Thermo Fisher Scientific, Waltham, MA, USA) according to the manufacturer’s protocol. Briefly, the pCAG-scR2agoTNF-Fc expression vector was transfected into Expi293F cells using ExpiFectamine 293 reagent. Seven days later, cultured medium was collected by centrifugation. The scR2agoTNF-Fc protein was recovered from the supernatant using KANEKA KanCapA (FUJIFILM Wako) and eluted with 0.1 M glycine–HCl (pH 2.8). Recovered protein was purified by size-exclusion chromatography using a HiLoad 16/600 Superdex 200 prep grade column (GE Healthcare, Piscataway, NJ, USA) in accordance with our previous report^21^.

### SDS-PAGE and immunoblotting

SDS-PAGE and immunoblotting were performed in accordance with our previous methods^21,24^. Briefly, samples were prepared with Laemmli buffer containing 5% 2-mercaptoethanol. SDS-PAGE was performed using Tris–glycine–SDS buffer (pH 8.3) and proteins were detected by Coomassie Brilliant Blue staining. For western blotting, proteins were transferred onto a PVDF membrane. After blocking nonspecific proteins, scR2agoTNF-Fc protein was probed with HRP-conjugated anti-human IgG antibody (Jackson ImmunoResearch Laboratories, West Grove, PA, USA). Visualization was performed by chemiluminescence with ECL Prime (GE Healthcare).

### Binding kinetics analysis

Binding kinetics were analyzed by SPR (BIAcore X-100, GE Healthcare)^24^. Fc fusion proteins of mouse TNFR2 and mouse TNFR1 (R&D Systems, Minneapolis, MN, USA) were immobilized on sensor chip CM5 by an amine coupling reaction. HBS-EP plus buffer (GE Healthcare) was used as running buffer. Proteins diluted with HBS-EP plus buffer were injected for 2 min at a flow rate of 30 μl/min, and dissociation was monitored for 2 min. The analysis was performed with single-cycle kinetics.

### Binding capacity of scR2agoTNF-Fc

LN cells were prepared from WT-B6 mice or TNFR2^−/−^B6 mice, respectively. Each cell type (5 × 10^5^ cells) was reacted with scR2agoTNF-Fc (0, 1, 10 ng/ml), anti-human IgG-Fc mAb/AlexaFluor 488, anti-mouse CD3 mAb/PE-Cy7 and anti-mouse CD4 mAb/PerCP for 1 h. Then, cells were probed with anti-mouse Foxp3 mAb/APC by intracellular staining using a Foxp3/transcription factor staining buffer set (Thermo Fisher Scientific). For assessing the binding capacity of scR2agoTNF-Fc, the rate of AlexaFluor 488-positive cells in CD3^+^CD4^+^Foxp3^+^ T cells was measured by FCM.

### In vitro cytotoxicity assay via TNFR2

Mouse TNFR2/Fas preadipocytes (1.5 × 10^4^ cells/well) were cultured with serially-diluted scR2agoTNF-Fc. After incubation for 48 h at 37 °C, cell viability was measured by a WST-8 colorimetric assay (Cell Counting Kit-8, Dojindo Laboratories, Kumamoto, Japan) according to the manufacturer’s protocol.

### Flow cytometry

Cells were suspended in PBS containing 2% FBS and stained with fluorescent-labeled anti-mouse surface molecule antibodies: CD4 (RM4-5)/PerCP, CD25 (PC61)/BV421, CD8 (53-6.7)/FITC, TNFR2 (TR75-89)/PE, and CD90.2 (30-H12)/PE (BioLegend, San Diego, CA). Live cells were stained by eBioscience Fixable Viability Dye eFluor 506 (Thermo Fisher Scientific). Anti-mouse CD16/CD32 (2.4G2) (BD Biosciences) was used for Fc blocking. For intracellular staining, anti-Foxp3 (FJK-16s)/APC (Thermo Fisher Scientific), anti-Foxp3 (ME-14)/BV421, anti-IL-4 (11B11)/PE, anti-Ki-67 (16A8)/PE (BioLegend), anti-IL-17(TC11-18H10)/PE (BD Biosciences), anti-phospho-IκB alpha (Ser32, Ser36) (RILYB3R)/eFluor 660 (Thermo Fisher Scientific), and anti-phospho-IKKα/β (Ser176/180) (16A6) /PE (Cell Signaling Technology) were used after fixation and permeabilization with an eBioscience Foxp3/Transcription Factor Staining Buffer Set (Thermo Fisher Scientific) according to the manufacturer’s protocol. Isotype controls matched for each antibody were used. For human molecule staining, anti-CD4 (RPA-T4)/APC (BioLegend), anti-Foxp3 (PCH101)/PE (eBioscience), and anti-TNFR2 (hTNFR-M1)/BV421 (BD Biosciences) were used. FCM was performed using a CytoFLEX cell analyzer (Beckman Coulter, Brea, CA). Data were analyzed using FlowJo software (BD Biosciences).

### Treg proliferation assay ex vivo

CD4^+^CD25^+^ Tregs or CD4^+^CD25^−^ Tconvs isolated from the LN of WT mice or TNFR2^−/−^ mice were labeled with CFSE (3 μM) using a CellTrace CFSE Cell Proliferation Kit (Thermo Fisher Scientific) for 5 min at room temperature. To compare the proliferation rates of molecules, CFSE-labeled cells (5 × 10^4^ cells/well) were cultured with soluble human IL-2 (10 U/ml) (Imunase35, Shionogi, Osaka, Japan), R2agoTNF (100 ng/ml), or scR2agoTNF-Fc (100 ng/ml) in a U-bottom 96-well plate for 72 h under stimulation of immobilized anti-mouse CD3ε mAb (0.5 μg/ml), which was immobilized for 16 h prior to the assay. To measure concentration dependency, CFSE-labeled cells were cultured with scR2agoTNF-Fc (1, 10, 100 ng/ml) under stimulation of immobilized anti-mouse CD3ε mAb in the same manner as above. After cell collection, cell division was measured by CFSE attenuation using FCM. For CFSE assays using whole CD4^+^ T cells, CD4^+^ T cells were prepared from the spleens of WT-B6 mice using an EasySep Mouse CD4^+^ T Cell Isolation Kit (VERITAS). Then, CD4^+^ T cells were labeled with CFSE (3 μM) as described above and CFSE-labeled CD4^+^ T cells (5 × 10^4^ cells/well) were cultured with or without scR2agoTNF-Fc (100 ng/ml) under stimulation of immobilized anti-mouse CD3ε mAb for 72 h. Next, cells were collected and immunostained for CD3, CD4, CD8, and Foxp3. Finally, cell proliferation was assessed by CFSE attenuation in CD3 gated CD4^+^Foxp3^+^ Tregs, CD4^+^Foxp3^−^ Tconvs, and CD8^+^ T cells using FCM.

### NF-κB signaling analysis

Intracellular signaling was assessed according to the phosphoflow method. LN cells (5 × 10^5^ cells/well) prepared from B6 mice were stimulated by scR2agoTNF-Fc (100 ng/ml) for 0, 5, 10, 30, or 60 min in a 96-well plate. After cell washing with PBS, cells were fixed and permeabilized with a Foxp3/transcription factor staining buffer set (Thermo Fisher Scientific). Cells were immunostained with anti-mouse CD3 mAb/PE-Cy7, anti-mouse CD4 mAb/PerCP, anti-CD8 mAb/FITC, anti-Foxp3 mAb/BV421, anti-phospho-IκB alpha (Ser32, Ser36) mAb/eFluor 660, and anti-phospho-IKKα/β mAb/PE for 1 h. Phospho-IκB-positive cells or phosphor-IKK-positive cells were detected in CD3 gated CD4^+^Foxp3^+^ Tregs, CD4^+^Foxp3^−^ Tconvs, and CD8^+^ T cells by FCM.

### Treg suppression assay

CD4^+^CD25^−^ Tconvs were purified from the LN of WT mice. CD4^+^CD25^+^ Tregs from WT- or TNFR2^−/−^ mice were purified separately from each LN. CD90.2^−^ cells were isolated from WT spleens as antigen-presenting cells (APCs). Cells were separated by a FACSAria cell sorter (BD Biosciences). CD4^+^CD25^−^ Tconvs were labeled with CFSE (3 μM) using a CellTrace CFSE Cell Proliferation Kit (Thermo Fisher Scientific) for 5 min at room temperature. CFSE-labeled Tconvs (5 × 10^4^ cells/well) were co-cultured with WT-Tregs or TNFR2^−/−^ Tregs at the desired ratio plus APCs (2 × 10^5^ cells/well) and purified anti-mouse CD3ε mAb (0.5 μg/ml) (145-2C11, BD Biosciences). Tregs were either pre-expanded with immobilized anti-mouse CD3 mAb and scR2agoTNF-Fc (100 ng/ml) for 72 h or stimulated by scR2agoTNF-Fc (1, 10, 100 ng/ml) in co-culture with Tconvs. After 72 h co-culture with Tregs and Tconvs, Tconv proliferation was estimated by CFSE dilution measured by FCM.

### Measurement of scR2agoTNF-Fc concentration in plasma

Male BALB/c mice (8-weeks-old) were injected i.p. with 30 μg R2agoTNF or scR2agoTNF-Fc. To investigate pharmacokinetics, blood was collected from the tail vein of mice at an appropriate interval after injection. Protein concentrations in plasma samples were measured by a human TNF ELISA kit (BD Biosciences) or human IgG ELISA kit (Bethyl Laboratories, Montgomery, TX, USA). The t½ and AUC were calculated by moment analysis.

### Treg expansion assay in vivo

scR2agoTNF-Fc (50 μg/mouse) was administered i.p. to WT mice twice a week for two weeks. Saline was administered i.p. as the negative control. Then, LN cells were prepared and stained with fluorescent-labeled antibodies for CD3(PE/Cy7), CD4(PerCP), CD8(FITC), and Foxp3(APC) as described in the FCM section. Rates of CD4^+^Foxp3^+^ Tregs, CD4^+^Foxp3^−^ Tconvs, and CD8^+^ T cells in CD3^+^ T cells from both groups were measured by FCM. The expressions of Ki-67, a proliferation marker, in CD4^+^Foxp3^+^ Tregs or CD4^+^Foxp3^−^ Tconvs were measured by FCM.

### FITC-induced contact hypersensitivity

Mice were sensitized epicutaneously at day 0 by applying 100 μl 0.5% (w/v) FITC diluted in a 4:1 mixture of acetone and dibutyl phthalate to the abdominal skin and challenged at day 5 by applying 20 μl 0.5% FITC onto the ear as previously described^56^. scR2agoTNF-Fc (5, 50 μg/mouse) was administered i.p. at day − 5, − 2, and + 1. Ear swelling and populations of CD4^+^Foxp3^+^ T cells and IL-4^+^ cells in the regional LN were assessed at 24 h after FITC challenge.

### Quantification of cytokine levels in ear tissues

Mice were sacrificed at the end of the study period. Ears from individual mice were isolated and homogenized with Cell Lysis Buffer M (FUJIFILM Wako) containing a complete protease inhibitor cocktail (Roche, Basel, Switzerland). Then, supernatant was collected by centrifugation. The concentration of mouse IL-4 in extracts was measured by ELISA (DuoSet, R&D Systems).

### Induction of arthritis and administration of scR2agoTNF-Fc

A mixed emulsion of bovine collagen type II (2 mg/ml, Chondrex, Redmond, WA, USA) and complete Freund’s adjuvant (CFA, 2 mg/ml, Chondrex) or incomplete Freund’s adjuvant (IFA, Chondrex) were prepared according to a standard CIA protocol by Chondrex, Inc. Then, 100 μl of the CFA emulsion was subcutaneously immunized to DBA/1 mice (male, 6-weeks-old) at the base of the tail, followed 3 weeks later by immunization with 100 μl IFA emulsion. scR2agoTNF-Fc and saline (negative control) were administered i.p. twice a week from day 22 after immunization.

### Assessment of inflammation and bodyweight

We had assessed the severity of arthritis by clinical score in previous report^24^. In a similar way, each paw was scored on a scale from 0–4 as follows: 0, normal paw; 1, mild but definite redness and swelling of the ankle or wrist or apparent redness and swelling limited to individual digits, regardless of the number of affected digits; 2, moderate redness and swelling of ankle or wrist; 3, severe redness and swelling of the entire paw including digits; and 4, maximally inflamed limb with involvement of multiple joints. The sum of the clinical scores of four paws was plotted as the degree of inflammation. Changes in bodyweight over the course of the experiment were measured.

### Human PBMC proliferation assay

A 96-well plate was pretreated with purified anti-human CD3ε mAb (0.5 μg/ml) (SK7, BioLegend). After 24 h and washing, human PBMCs (5 × 10^4^ cells/well) (Precision for Medicine, Bethesda, MD, USA) were cultured with R2-7 (100 ng/ml) or scR2-7-Fc (100 ng/ml). After 72 h, the population of CD4^+^Foxp3^+^ Tregs and CD4^+^Foxp3^−^ Tconvs were measured by FCM. Fluorescent-labeled antibodies used for cell staining are described in the FCM section. The study followed the relevant Ministry of Health, Labor and Welfare of Japan guidelines.

### Statistical analysis

The significance of differences was determined by one-way analysis of variance (ANOVA) followed by a secondary test (Tukey) or unpaired Student’s *t*-test. *p < 0.05, **p < 0.01, ***p < 0.001 were considered statistically significant. All statistical analyses were performed using GraphPad Prism for Windows Software version 6.0 (GraphPad Software, San Diego, CA, USA).

### Supplementary Information

Supplementary Figures.

## Data Availability

The datasets generated during the current study are available at DDBJ accession numbers LC739729 (scR2agoTNF-Fc: https://getentry.ddbj.nig.ac.jp/getentry/na/LC739729) or LC739854 (scR2l-7-Fc: pending publication).
