# Supplementary material for: Bivalent structure of a TNFR2-selective and agonistic TNF-α mutein Fc-fusion protein enhances the expansion activity of regulatory T cells
Source: Sci Rep. 2023 Aug 23;13:13762. doi: 10.1038/s41598-023-40925-9 (PMC10447426; doi:10.1038/s41598-023-40925-9)
Supplement: Supplementary file 1 — Supplementary Figures. [file 41598_2023_40925_MOESM1_ESM.pdf]

Supplementary Fig. S1

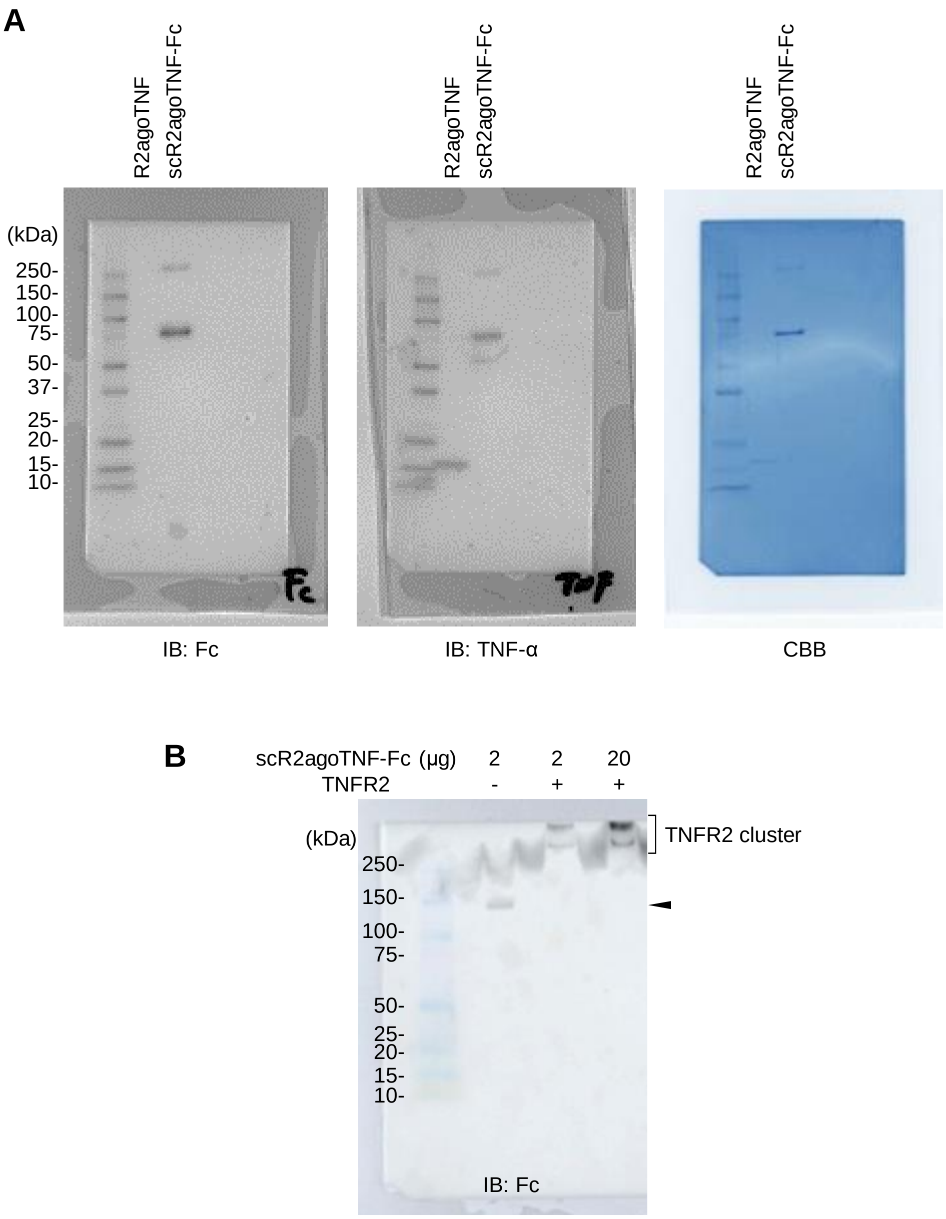

Supplementary Fig. S1 Full-length blots in Fig. 1D and 1H
(A) Original blots of Fig. 1D. (B) Original blot of Fig. 1H.

Supplementary Fig. S2

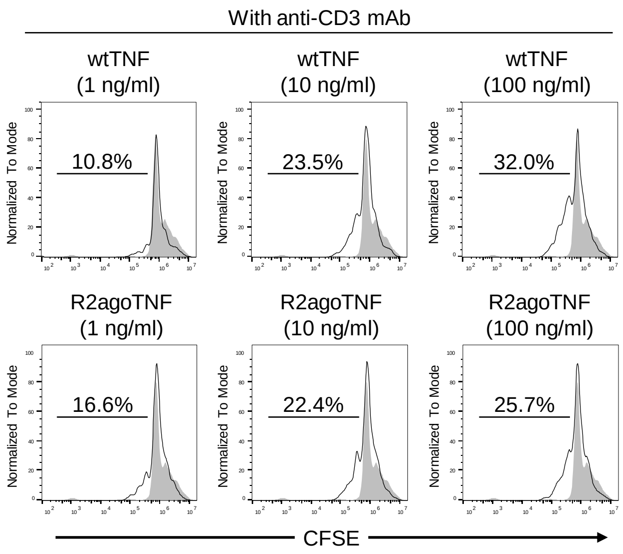

Supplementary Fig. S2 *Ex vivo* Treg expansion by wtTNF and R2agoTNF.

$CD4^+CD25^+$ Tregs isolated from the LNs of WT mice were labeled with CFSE (3 μM) using a CellTrace CFSE Cell Proliferation Kit (Thermo Fisher Scientific). To compare the proliferation rates of molecules, CFSE-labeled Tregs (5×10^4 cells/well) were cultured with soluble wtTNF (1, 10 and 100 ng/ml) or R2agoTNF (1, 10 and 100 ng/ml) in a U-bottom 96-well plate for 72 h under stimulation with immobilized anti-mouse CD3ε mAb (0.5 μg/ml). After cell collection, cell division was measured by CFSE attenuation using FCM. Both wtTNF and R2agoTNF expand Tregs *ex vivo* in a concentration-dependent manner.

Supplementary Fig. S3

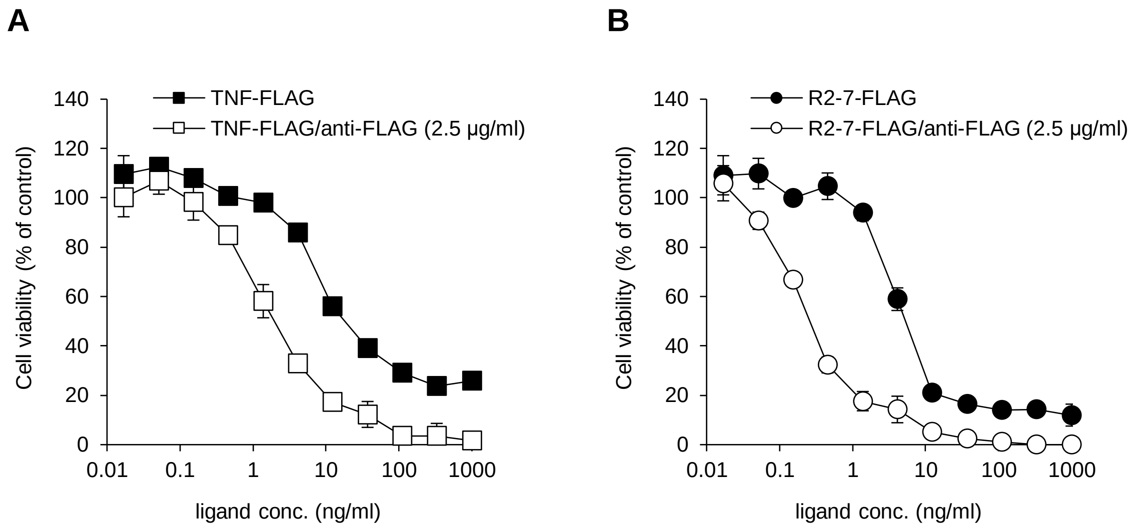

Supplementary Fig. S3 Enhancing TNFR2 agonist activity by dimerization or multimerization.
Human TNFR2/Fas preadipocytes (1.5×10^4 cells/well) were cultured with serially-diluted recombinant human TNF-FLAG (A) or R2-7-FLAG (B) with or without anti-FLAG-tag mAb (2.5 μg/ml). The antibody was added for dimerization or oligomerization of FLAG-tagged ligand. After incubation for 48 h at 37℃, cell viability was measured by a WST-8 colorimetric assay (Cell Counting Kit-8, Dojindo Laboratories). Both TNF-FLAG and R2-7-FLAG induced cell death in a concentration-dependent manner. Furthermore, anti-FLAG-tag mAb enhanced the cytotoxicity.

Supplementary Fig. S4

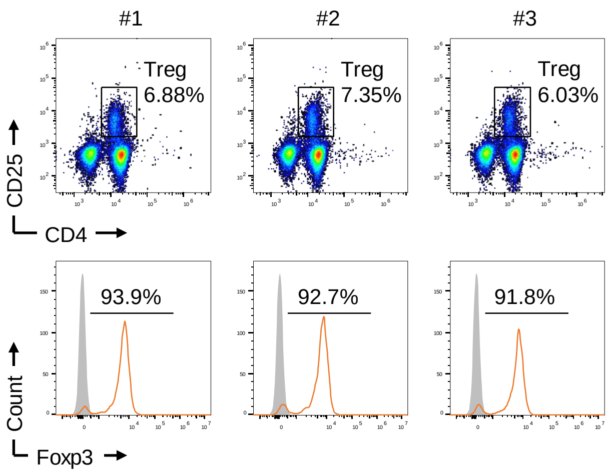

Supplementary Fig. S4 Foxp3 expression in CD4⁺CD25⁺ Tregs

After cell preparation from lymph nodes of C57BL/6 (n=3), cells (1×10^6 cells) were stained with fluorescent-labeled anti-mouse molecule antibodies: CD4 (RM4-5)/PerCP, CD25 (PC61)/BV421 and Foxp3 (FJK-16s)/APC. The Foxp3/Transcription Factor Staining Buffer Set (Thermo Fisher Scientific) was used for staining according to the manufacturer's protocol. The cell population was measured by FCM. Histograms show Foxp3 expression level in $CD4^+CD25^+$ Tregs. More than 90% of $CD4^+CD25^+$ Tregs were $Foxp3^+$ cells.
